# Medical Adhesive-Related Skin Injury Associated with Surgical Wound Dressing among Spinal Surgery Patients: A Cross-Sectional Study

**DOI:** 10.3390/ijerph18179150

**Published:** 2021-08-30

**Authors:** Jeounghee Kim, Yongsoon Shin

**Affiliations:** 1 Department of Nursing, Asan Medical Center, Seoul 05505, Korea; jeounghee@amc.seoul.kr; 2 College of Nursing, Hanyang University, Seoul 04763, Korea

**Keywords:** adhesives, nursing, postoperative care, skin injury, surgical tape

## Abstract

The aim of this cross-sectional study was to determine the incidence, types, and factors associated with medical adhesive-related skin injuries (MARSIs) among spinal surgery patients. Adult patients who underwent planned spinal surgery under general anesthesia at a tertiary hospital in Seoul, Korea were enrolled. Data were collected from March through April 2019. Skins under surgical wound dressings were evaluated for MARSI once every morning until discharge. Skin injuries lasting for 30 min or more were considered as MARSIs. Logistic regression was performed to identify factors associated with MARSI. The incidence of MARSIs in surgical areas was 36.4% and the rate per 100 medical adhesives was 9.8%. All MARSIs occurred on postoperative day 1 or 2. A history of contact dermatitis (OR = 10.517, 95% CI = 3.540–31.241, *p* < 0.001) and late ambulation (OR = 1.053, 95% CI = 1.012–1.095, *p* = 0.010) were identified as risk factors for MARSI. Spinal surgery patients were at high risk of MARSIs associated with surgical wound dressings. Patients with a history of contact dermatitis or prolonged bed rest periods need more active skin assessment and more careful skin care to prevent MARSIs after spinal surgery.

## 1. Introduction

Medical adhesives are required for surgical site dressings and to facilitate the fixation of intravenous injections, central lines, and various catheters and monitors during surgery. Peeling off adhesive dressings removes loosely bound epidermal cells and stratum corneum, thereby compromising the skin barrier and increasing transepidermal water loss, which ultimately results in skin breakdown and medical adhesive-related skin injuries (MARSIs) [1]. MARSI is a consensus statement on 23 assessment, prevention, and treatment as defined by the MARSI Consensus Group of McNichol et al. and refers to erythema and skin anomalies (e.g., blisters, erosion, cracks) that last for more than 30 min after the removal of a medical adhesive [2]. Even if there is no noticeable trauma, the removal of adhesives typically results in varying degrees of surface epidermal cell layer separation, and repeated application and removal reduces epidermal thickness [3]. MARSIs include mechanical skin injury caused by epidermal skin stripping resulting in skin tears, tension injuries, blisters, irritant contact, allergic dermatitis, folliculitis, and moisture-associated skin damage [4].

Although MARSI is a complication that affects patients of all ages [2], the elderly represent a particularly vulnerable population due to changes in the structural and functional properties of the skin as well as its neurosensory perception, permeability, response to damage, repair capacity, and increases in the incidence of some skin diseases [5]. When MARSIs occur, the patient experiences unexpected pain and tissue trauma, leading to reversible or irreversible skin damage, increased medical expenses, and decreased quality of life [6]. Accordingly, the awareness of MARSI and the related burden has increased in recent years [7], and studies to date have reported an incidence of 25% in medical-surgical wards [8], 11% among critically ill patients [9], and 20% of surgical dressings [10].

Spinal surgery often requires the process of attaching and removing medical adhesives for a variety of purposes, including monitor probes, catheter fixation, draping, and wound disinfection. In addition, many patients consequently experience MARSIs and suffer from discomfort and pain in addition to their major surgical wounds. Skin assessment, including of MARSIs, is a critical nursing task that strongly affects patient outcomes after spinal surgery, and strategies should be established to maximize patient safety and minimize damage [11]. However, the characteristics of MARSIs in surgical wound dressing after spinal surgery have not been fully established, and few risk factors for MARSI have been evaluated in patients undergoing spinal surgery.

Therefore, this study aimed to identify the incidence, types, and risk factors associated with MARSI related to surgical wound dressing among spinal surgery patients.

## 2. Materials and Methods

### 2.1. Setting and Participants

This cross-sectional study sought to determine the incidence of and risk factors for MARSI in patients with spinal surgery. From March through April 2019, a total of 143 people were enrolled in neurosurgery clinics at Asan Medical Center, a tertiary hospital in Seoul, Republic of Korea. The inclusion criteria were patients who were 20 years of age or older who underwent planned surgery under general anesthesia due to spinal disease. Patients with disorientation, communication difficulties, or prior radiotherapy at the surgical site were excluded.

### 2.2. Variables and Measurement

The skin was directly assessed for MARSI within 10 min before and after removing medical adhesive surgical site dressings, and skin injuries lasting for 30 min or more were considered MARSIs. We calculated 2 types of incidence rates for MARSI as follows using the same formula used in Kim et al. [12]: (1) rate per 100 patients = number of patients with MARSIs/number of patients with non-MARSIs × 100, (2) incidence rate per 100 medical adhesives = number of MARSIs/number of medical adhesives × 100. Types of MARSIs included epidermal stripping, tension injuries or blisters, skin tears, contact dermatitis, maceration, and folliculitis [13]. The overall skin condition of all subjects, including around the wound, was classified and documented by the researcher.

### 2.3. Data Collection and Procedure

Patients were enrolled in the study upon providing written informed consent after admission to the hospital, typically 1 day before their scheduled operation date. Careful removal of all adhesives was performed by one researcher to minimize the risk of skin injury. Perioperatively, surgical drapes (3M™ Loban™ 2 Antimicrobial Incise Drape-6650, 3M Health Care, Saint Paul, MN, USA) were removed and povidone-iodine (Betadine topical solution) disinfection was applied with a pressure dressing, gauze, and a fixing roll (unwoven fabric adhesive plaster). From the first day after surgery, the compression dressing was removed and the wound area was disinfected with 0.5% chlorhexidine once a day. After disinfection, the dressing was applied with Meditouch Border (soft silicone adhesive type polyurethane foam dressing, Ildong Pharmaceutical, Seoul, Korea). To minimize skin damage, a clinical nurse specialist carefully removed all adhesives by keeping the tape parallel to the skin and removing the adhesive in a “low and slow” manner. Patients were instructed to initiate ambulation on the day after surgery, and discharge was planned for the second day after surgery. Study procedures were reviewed and approved by the institutional review board of Asan Medical Center (approval #2018-1043).

### 2.4. Data Analysis

The data were analyzed using SPSS Statistics for Windows, version 22.0 (IBM Corp., Armonk, NY, USA). Continuous variables with normal distributions are presented as mean ± standard deviation (SD). If homogeneity of variance was confirmed, the independent-samples *t*-test was used for intergroup comparisons. The chi-square test or Fisher’s exact test was used for categorical data, as appropriate. Binary logistic regression analysis and multivariate analysis with logistic LR forward stepwise regression were used to determine factors affecting MARSI incidence. Statistical significance was defined as a *p*-value < 0.05.

## 3. Results

### 3.1. Patient Characteristics

Of the 143 participants, women accounted for 53.8%, and the mean age was 63.7 ± 12.1 years; 16.8% had contact dermatitis, and 17.5% had diabetes (Table 1). The most common diagnosis was spinal stenosis (67.9%) and the mean operation time was 291.4 ± 104.0 min. The average time from surgery to the first ambulation was 22.6 ± 9.3 h.

### 3.2. Comparison of Patient Characteristics According to MARSI Features

The differences between the MARSI and non-MARSI groups are shown in Table 1. The prevalence of contact dermatitis was significantly higher among patients who experienced MARSIs than among those who did not (χ^2^ = 22.83, *p* < 0.001). The MARSI group had a longer delay to the first ambulation after surgery compared with the non-MARSI group (χ^2^ = −2.29, *p* = 0.025). Postoperative serum protein (t = 2.55, *p* = 0.012) and albumin (t = 2.28, *p* = 0.024) levels were significantly different between the MARSI and non-MARSI groups.

### 3.3. Incidence and Characteristics of MARSI

**Figure 1:**
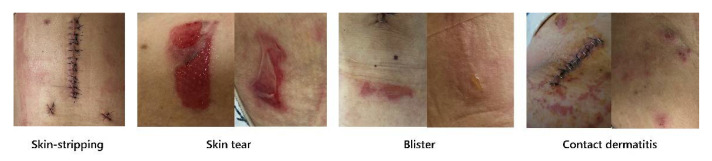
Different types of MARSI.

Among 143 patients, 52 patients developed MARSIs (overall incidence rate: 36.4%) and the rate per 100 medical adhesives was 9.83% (Table 2). Contact dermatitis was the most common type of MARSI (34.6%), followed by skin tear (28.8%), skin-stripping (23.1%), and tension injury or blister (13.5%). MARSIs occurred on day 1 after surgery in more than half (51.9%) of the cases and on day 2 after surgery in 48.1% (Figure 1).

### 3.4. Factors Affecting MARSI

On logistic regression analysis (Table 3), a history of contact dermatitis (OR = 10.517, 95% CI = 3.540–31.241, *p* < 0.001) and longer time to the first ambulation (OR = 1.053, 95% CI = 1.012–1.095, *p* = 0.010) were shown to be significantly associated with the occurrence of MARSI.

## 4. Discussion

This study was conducted to evaluate the incidence of MARSI and to identify factors associated with MARSI occurrence after spinal surgery. The use of adhesive wound dressings is essential during standard treatment among spinal surgery patients. Adhesive-related skin damage often occurs in such patients due to the use of glue for frequent firm fixation, removal of coarse adhesives, and repeated use of adhesives in the process of wound disinfection [14]. Such injuries are associated with discomfort, pain, additional treatment, and increased medical expenses.

In this study, the incidence of MARSI associated with surgical wound dressing after spinal surgery was 36.4%. The incidence of surgical site MARSI per 100 patients in this study was higher than those of previous studies, which were reported to be 11% to 22.7% in critically ill patients [9,14,15,16] and 27.9% in hospitalized patients [17]. A systematic review [18] reported that the incidence of MARSI in the intensive care unit was as high as 41.2%. In this study, the incidence rate per 100 medical adhesives was 9.83%, which was higher than the 8.5% observed rate in critically ill pediatric patients [12]. A recent study reported that 20% of MARSIs in intensive care units were associated with sterile surgical dressings [10]. The incidence of medical adhesive-related skin injuries in this study was higher than the overall incidence in other studies, suggesting that surgical wound dressings may be the cause of MARSIs.

While previous studies investigated the incidence of medical adhesive-related skin injuries in all areas, this study was limited to spinal surgical wounds, so there may be differences in the incidence rate. However, in this study, the researcher directly checked the skin condition of all patients before applying the adhesive; after that, skin condition was assessed daily when changing the wound dressing, such that the occurrence date of MARSI and the reporting date coincided.

We found that factors affecting MARSI occurrence were a history of contact dermatitis and longer time to the first ambulation after surgery. Patients with contact dermatitis were 10.5 times more likely to develop a MARSI than those without; this is consistent with a recent report [7], which reported that underlying medical conditions can affect the skin, and that dermatological conditions themselves increase the risk of medical adhesive-related skin damage. Similarly, another study [1] reported that a history of contact dermatitis was a risk factor for contact dermatitis among MARSIs of the peripherally inserted central catheter insertion site in cancer patients.

When replacing dressings, soft unwoven fabrics are most frequently associated with pain and skin tearing, and soft silicone adhesives are least frequently associated with pain and tearing [19]. Soft silicone adhesives create many contact points on the uneven surface of the skin, thereby providing a safe level of adhesion; the development of modern wound dressing materials has noticeably mitigated dressing-associated damage to the stratum corneum [20,21]. However, in the case of patients with contact dermatitis history, special attention is required regardless of the adhesive product used.

A longer delay for the first ambulation after spinal surgery was associated with a higher likelihood of MARSI occurrence. As bed rest time increased, skin integration at the surgical site changed. In addition, 87.3% of the patients underwent cervical, thoracic, and lumbar surgery with a posterior approach. The thickness of the skin varies depending on the anatomical location. For example, the back and forehead are more vulnerable than the abdomen or forearm and are thus more susceptible to skin-stripping during adhesive removal [22]. Spinal surgery requires a period of stabilization after surgery due to the instability of the spine and pain at the surgical site. Prolonging this period or delaying first postoperative ambulation can cause changes in skin integrity, pH, temperature, and humidity at the wound site [23], while the patient is wearing a pressure-adhesive dressing. For these reasons, the incidence of MARSIs at surgical wound dressing sites was high in this study.

Long-term moisture accumulation under adhesive tapes or dressings has been known as a cause of maceration, one type of MARSI [7]. In this study, most of the patients’ dressings were not soaked, except for a small amount of oozing in some of the dressings on the first day after surgery. The site of occurrence of MARSI was not the incision site, but the area where the tape was applied. Therefore, it is difficult to conclude that excessive exudate contributes to the incidence of MARSI.

Skin tearing is more common among the elderly, and the average age of the participants in this study was 63.7 years. Older adults have less collagen elastin, less fatty tissue, more wrinkles due to reduced skin elasticity, fewer sebum glands, drier skin due to sweat gland inactivity, and their skin is more vulnerable to normal wear and tear [24,25]. Old age has been reported as a risk factor for MARSI among cancer patients [1], but the incidence of MARSI in this study was not significantly associated with age; therefore, further studies on this topic are needed.

Lower postoperative serum protein and albumin concentrations were significantly associated with the incidence of MARSI in univariate analysis. Malnutrition is a major risk factor for skin integration disorders [25,26]. Energy, carbohydrates, proteins, fats, vitamins, and minerals can all affect skin integration and healing processes. Serum albumin levels have been identified as an independent risk factor for skin breakdown [25,27]. Even if the nutritional status of the patient before surgery is good, serum protein and albumin may rapidly decrease after surgery and increase the risk of MARSIs. However, since albumin and protein levels were not identified as significant factors in logistic regression analysis, follow-up studies are needed to provide more information.

## 5. Limitation and Strengths

As a single-institution study, this study has limitations in terms of generalization to spinal surgery patients in different settings. In addition, the process of applying and removing surgical drapes in the operating room was not sufficiently controlled, and the follow-up duration was relatively short. Moreover, a group that is heterogeneous in terms of the location of the operation and the type of procedure may have influenced the results. Nevertheless, our study is novel and potentially valuable in that we elucidated the characteristics of MARSI in the surgical wound dressing area and identified its risk factors in patients with spinal surgery.

## 6. Conclusions

Spinal surgery patients were at high risk of MARSI associated with surgical wound dressings. To prevent such injuries, early ambulation after spinal surgery should be encouraged, and caution should be used when dressing wounds in patients with a history of contact dermatitis.

## Figures and Tables

**Table 1:** Participant characteristics. (*n* = 143).

Variables	Categories	Total (*n* = 143)	MARSI (*n* = 52)	Non-MARSI (*n* = 91)	χ^2^ or t	*p*
		*n* (%) or M ± SD	*n* (%) or M ± SD	*n* (%) or M ± SD		
Sex	Male	66 (46.2)	21 (40.4)	45 (49.5)	1.09	0.383
	Female	77 (53.8)	31 (59.6)	46 (50.5)		
Age (year)		63.7 ± 12.1	63.2 ± 12.7	64.0 ± 11.8	0.42	0.674
BMI		24.9 ± 4.9	24.9 ± 3.8	25.0 ± 5.4	0.06	0.955
Alcohol use	Yes	39 (27.3)	12 (23.1)	27 (29.7)	0.73	0.440
Smoking	Yes	24 (16.8)	6 (11.5)	18 (19.8)	1.61	0.250
Comorbidity	Cardiovascular	57 (39.9)	25 (48.1)	32 (35.2)	2.30	0.156
	Endocrinology	25 (17.5)	10 (19.2)	15 (16.5)	0.17	0.819
	Cerebrovascular	8 (5.6)	4 (7.8)	4 (4.3)	0.76	0.304
	Chronic disease *	23 (16.1)	5 (9.8)	18 (19.6)	2.32	0.097
Steroid use Hx	Yes	6 (4.2)	2 (3.9)	4 (4.3)	0.02	0.635
Contact dermatitis Hx	Yes	24 (16.8)	19 (36.5)	5 (5.5)	22.83	<0.001
Spine operation Hx	Yes	47 (32.9)	22 (42.3)	25 (27.5)	3.30	0.095
Anticoagulation drug Hx	Yes	32 (22.4)	15 (28.8)	17 (18.7)	1.97	0.211
Diagnosis	HNP	10 (7.0)	3 (5.9)	7 (7.6)	5.98	0.309
	Stenosis	97 (67.8)	38 (74.5)	59 (64.1)		
	Benign cord tumor	16(11.2)	3 (5.9)	13 (14.1)		
	Spine metastsis	8 (5.6)	1 (2.0)	7 (7.6)		
	Fracture	5 (3.5)	3 (5.9)	2 (2.2)		
	Others	7 (4.9)	3 (5.9)	4 (4.3)		
Posture during surgery	Supine	20 (14.0)	6 (11.8)	14 (15.2)	0.33	0.382
	Prone	123 (86.0)	45 (88.2)	78 (84.8)		
Surgical site	Anterior cervical	16 (11.2)	4 (7.8)	12 (13.0)	3.74	0.442
	Posterior cervical	14 (9.8)	7 (7.6)	7 (7.6)		
	Posterior thoracic	24 (16.8)	6 (11.8)	18 (19.6)		
	Posterior lumbar	85 (59.4)	32 (62.7)	53 (57.6)		
	Others	4 (2.8)	2 (3.9)	2 (2.2)		
Extent of surgery	1 level	69 (48.3)	20 (39.2)	49 (53.3)	3.49	0.175
	2 level	52 (36.4)	20 (39.2)	32 (34.8)		
	3 level or more	22 (15.4)	11 (21.6)	11 (12.0)		
Duration of operation (min)		291.37 ± 103.97	312.17 ± 109.41	279.48 ± 99.39	−1.82	0.070
Time to the first ambulation after the surgery (hours)		22.59 ± 9.27	25.10 ± 10.83	21.16 ± 7.97	−2.29	0.025
Time to the first surgical wound dressing (hours)		21.13 ± 5.44	22.17 ± 6.40	20.53 ± 4.74	−1.75	0.082
Preop clinical data	Protein (g/dL)	6.99 ± 0.53	6.95 ± 0.58	7.01 ± 0.50	0.610	0.547
	Albumin (g/dL)	3.87 ± 0.38	3.86 ± 0.43	3.88 ± 0.35	0.36	0.721
	Hemoglobin (g/dL)	13.39 ± 1.73	13.27 ± 1.74	13.45 ± 1.73	0.58	0.562
	WBC (×10^2^/μL)	6.56 ± 1.82	6.31 ± 1.41	6.70 ± 2.00	1.33	0.185
Postop clinical data	Protein (g/dL)	5.63 ± 0.69	5.44 ± 0.76	5.74 ± 0.62	2.55	0.012
	Albumin (g/dL)	3.11 ± 0.45	3.00 ± 0.49	3.17 ± 0.41	2.28	0.024
	Hemoglobin (g/dL)	11.45 ± 2.20	11.04 ± 2.38	11.69 ± 2.06	1.69	0.092
	WBC (×10^2^/μL)	8.81 ± 3.65	8.60 ± 3.62	8.92 ± 3.68	0.51	0.610

MARSI: medical adhesive-related skin injury; BMI: body mass index; Hx: History; HNP: herniated nucleus pulpous; Preop: preoperative; Postop: postoperative; WBC: white blood cell. * Includes cancer, thyroid disease, and benign prostatic hyperplasia.

**Table 2:** Incidence and characteristics of MARSI. (*n* = 143).

Variables	Categories	*n* (%)
Incidence of MARSI	Rate per 100 patients	52 (36.4)
	Rate per 100 medical adhesives	9.83%
MARSI type (*n* = 52)	Skin-stripping	12 (23.1)
	Skin tear	15 (28.8)
	Tension injury or blister	7 (13.5)
	Contact dermatitis	18 (34.6)
Time to the occurrence of MARSI (days) (*n* = 52)	Postoperative day 1	27 (51.9)
	Postoperative day 2	25 (48.1)

MARSI: medical adhesive-related skin injury.

**Table 3:** Factors associated with the occurrence of MARSI. (*n* = 143).

Variables	B	SE	Wald Statistic	OR	95% CI	*p*
Contact dermatitis history	2.353	0.555	17.943	10.517	3.540–31.241	<0.001
Duration of operation (min)	0.001	0.002	0.334	1.001	0.991–1.006	0.563
Time to the first ambulation (h)	0.052	0.020	6.651	1.053	1.012–1.095	0.010
Post OP protein (g/dL)	−0.417	0.457	0.834	0.659	0.269–1.613	0.361
Post OP albumin (g/dL)	0.112	0.708	0.025	1.119	0.279–4.484	0.874

MARSI: medical adhesive-related skin injury; OR: odds ratio; CI: confidence interval; OP: Operation.

## Data Availability

The data presented in this study are available on request from the corresponding author.
